# [^18^F]FSPG-PET provides an early marker of radiotherapy response in head and neck squamous cell cancer

**DOI:** 10.1038/s44303-024-00038-y

**Published:** 2024-08-09

**Authors:** Khrishanthne Sambasivan, Will E. Tyrrell, Rizwan Farooq, Jenasee Mynerich, Richard S. Edwards, Muhammet Tanc, Teresa Guerrero Urbano, Timothy H. Witney

**Affiliations:** 1 School of Biomedical Engineering and Imaging Sciences, King’s College London, London, UK; 2 Department of Clinical Oncology, Guy’s and St Thomas’ NHS Foundation Trust, London, UK; 3 Faculty of Dentistry, Oral & Craniofacial Sciences and School of Cancer & Pharmaceutical Sciences, King’s College London, London, UK

## Abstract

The ability to image early treatment response to radiotherapy in head and neck squamous cell carcinoma (HNSCC) will enable the identification of radioresistant tumor volumes suitable for treatment intensification. Here, we propose the system x_c_^−^ radiotracer (4*S*)-4-(3-[^18^F]fluoropropyl)-L-glutamate ([^18^F]FSPG) as a non-invasive method to monitor radiation response in HNSCC. We assessed temporal changes in cell death, antioxidant status, and [^18^F]FSPG retention following a single dose of 10 Gy irradiation in FaDU HNSCC cells. Next, using a fractionated course of radiotherapy, we assessed tumor volume changes and performed [^18^F]FSPG-PET imaging in FaDU-bearing mouse xenografts, followed by ex vivo response assessment. In cells, 10 Gy irradiation reduced [^18^F]FSPG retention, coinciding with the induction of apoptosis and the production of reactive oxygen species. In vivo, [^18^F]FSPG tumor retention was halved seven days after the start of treatment, which preceded radiotherapy-induced tumor shrinkage, thereby confirming [^18^F]FSPG-PET as an early and sensitive marker of radiation response.

Radiotherapy is the treatment of choice for early-stage/locally advanced head and neck squamous cell carcinoma (HNSCC) when organ preservation is preferred, or surgery is not an option. Unfortunately, due to radio-resistance, locoregional recurrence occurs in 10–50% of cases, depending on the tumor site and stage^1^. Radical radiotherapy for locally advanced HNSCC consists of daily treatment for a period of six to seven weeks and is associated with multiple short- and long-term toxicities^2^. Patients whose tumors do not respond to radiotherapy need to be identified early on during treatment so that they might be considered for radiotherapy dose escalation^3^, addition of hypoxia modification therapy^4^, or even a switch to surgical resection^5^. To maximize the potential of these novel treatment strategies, biomarkers to predict radioresistance or monitor early response are pivotal to facilitate an individualized treatment plan.

One successful strategy used to monitor the efficacy of systemic anti-cancer therapy is the imaging of tumor redox status with (4*S*)-4-(3-[^18^F]fluoropropyl)-L-glutamic acid ([^18^F]FSPG; Supplementary Fig. 1A)^6–11^. Cancer cells experience elevated levels of intracellular ROS and upregulated antioxidant production, such as glutathione (GSH), to circumvent this oxidative stress^12^. [^18^F]FSPG provides a surrogate marker of GSH utilization through imaging the amino acid transporter system x_c_^−6^, which delivers intracellular cystine for *de novo* GSH biosynthesis. In animal models of ovarian cancer, tumor-associated [^18^F]FSPG retention was reduced following treatment with doxorubicin, which preceded tumor volume changes and correlated with the degree of oxidative stress within the cell^6^. Importantly, [^18^F]FSPG has been successfully used for cancer imaging in clinical trials, including in HNSCC patients^13,14^. Given that radiotherapy induces extensive oxidative stress^15^, and the promising ability of [^18^F]FSPG to monitor this response to therapy, we sought to establish if [^18^F]FSPG could monitor early and localized radiation response in HNSCC.

## Results

### 10 Gy irradiation induces apoptosis, accompanied by oxidative stress in FaDU cells

**Fig. 1:**
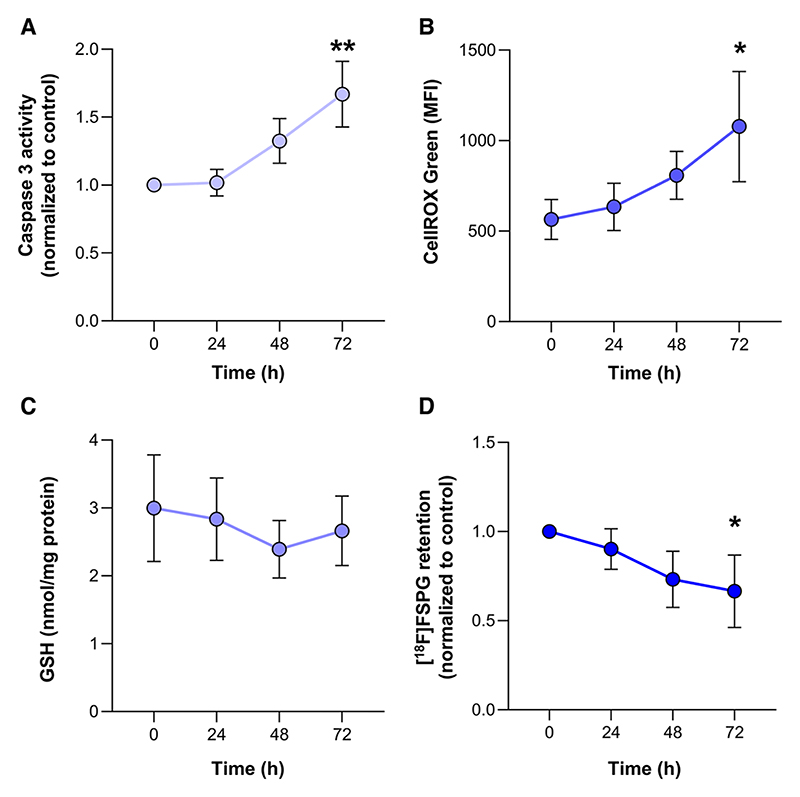
10 Gy Radiation induces apoptosis and oxidative stress at 72 h, which coincides with a reduction in [^18^F]FSPG retention. **A** Apoptosis was assessed through the measurement of caspase-3 activity. **B** Flow cytometric measurement of total ROS levels using CellROX Green. **C** Changes in intracellular GSH following radiotherapy. **D** Time course of [^18^F]FSPG retention following radiotherapy. Data are presented as mean ± SD for *n* = 3 biological replicates. *p < 0.05; **p < 0.001.

A single dose of 10 Gy was chosen to induce cell death in FaDU cells. The time course of apoptosis and oxidative stress (quantified by ROS and GSH levels, respectively) were measured up to 72 h at this dose (Fig. 1). There was a temporal increase in apoptotic cell death between 24 and 72 h after treatment (*p* < 0.001, *n* = 3, Fig. 1A), which coincided with a 91% increase in ROS levels (*p* < 0.05, *n* = 3, Fig. 1B). GSH levels remained unchanged over the entire time course (*p* = 0.19, *n* = 3, Fig. 1C).

### [^18^F]FSPG retention in cells is reduced following radiotherapy treatment

[^18^F]FSPG tumor retention is decreased following exposure to drugs that induce oxidative stress^6^. In keeping with this finding, [^18^F]FSPG retention in FaDU cells grown in culture was reduced by 33% 72 h after 10 Gy irradiation, falling from 11.1 ± 2.4% radioactivity/mg protein in untreated cells to 7.4 ± 2.6% radioactivity/mg protein after treatment, respectively (*p* < 0.05, *n* = 3, Fig. 1D and Supplementary Fig. 1B). A similar response was observed at both 4 and 8 Gy 96 h after irradiation (Supplementary Fig. 1C).

### [^18^F]FSPG-PET detects radiotherapy response in FaDU xeno-grafts before tumor shrinkage

**Fig. 2:**
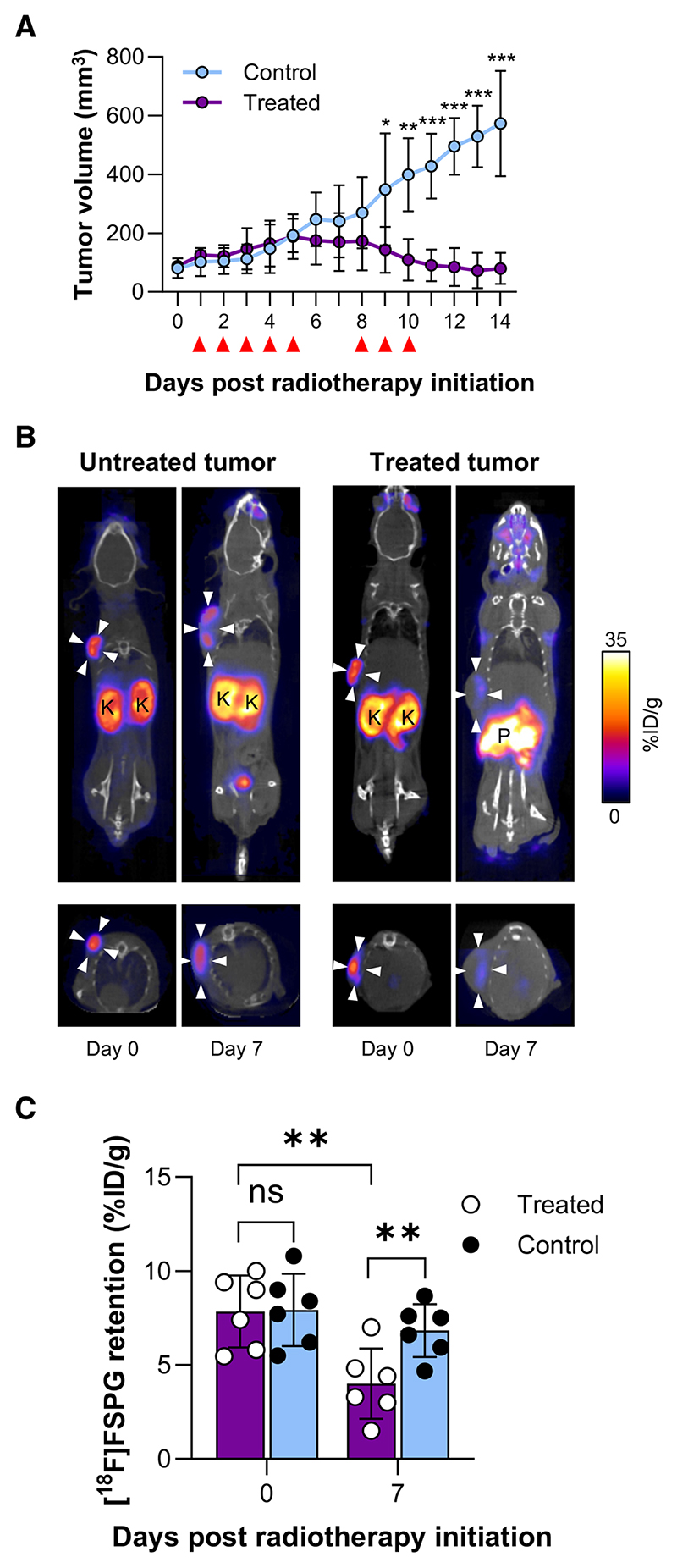
[^18^F]FSPG-PET is an early marker of radiation response in FaDU tumors. **A** Tumor volume changes following treatment with 24 Gy in 8 fractions of radio-therapy. Red triangles indicate 3 Gy radiotherapy treatment. **B** Representative PET/CT coronal and axial single slice images in control and radiotherapy-treated tumour-bearing mice 40–60 min post-injection. White arrowheads indicate the tumor margins. Images shown are on day 0 (pre-treatment) and day 7 after 5 fractions/15 Gy of radiotherapy. **C** Mean [^18^F]FSPG retention in treated and control cohorts at day 0 and 7. ns not significant; *p < 0.05; **p < 0.001; ***p < 0.0001; *n* =6 mice per group.

We next asked whether [^18^F]FSPG could monitor radiation response in vivo using PET imaging. FaDU tumor xenograft-bearing mice were treated with a fractionated dose of radiotherapy which was previously shown to be efficacious^16^. Following an initial increase in tumor volume, radiotherapy-induced cytostasis between days 5 and 8, with tumors shrinking over the remainder of the 14-day time course. Conversely, there was a linear increase in tumor volume in untreated tumors (Fig. 2A).

The mice bearing FaDU xenografts underwent [^18^F]FSPG-PET scans at day 0 and 7. Day 7 was selected as the imaging time point as it preceded tumor shrinkage whilst maximizing the delivered radiation dose. Prior to treatment, [^18^F]FSPG distribution was characterized by high tumor retention (~8% ID/g), with background activity noted only in the pancreas, kidney, and bladder (Fig. 2B)^6^. [^18^F]FSPG tumor retention was well-matched in animals randomly selected by tumor size for either treatment or as controls (7.8 ± 1.9% ID/g vs. 7.9 ± 1.9% ID/g, respectively, *n* = 6, *p* = 0.94). By day 7, [^18^F]FSPG retention was halved in the treated cohort, falling from 7.8 ± 1.9% ID/g to 4.0 ± 1.9% ID/g (*p* = 0.004, *n* = 6, Fig. 2B, C). At this time, [^18^F]FSPG tumor retention remained unchanged in control tumors (7.9 ± 1.9% ID/g and 6.8 ± 1.4% ID/g at day 0 and 7, respectively, *p* = 0.32, *n* = 6). The decrease in [^18^F]FSPG retention in the treated tumors preceded a reduction in tumor volume, which was observed on day 9.

**Fig. 3:**
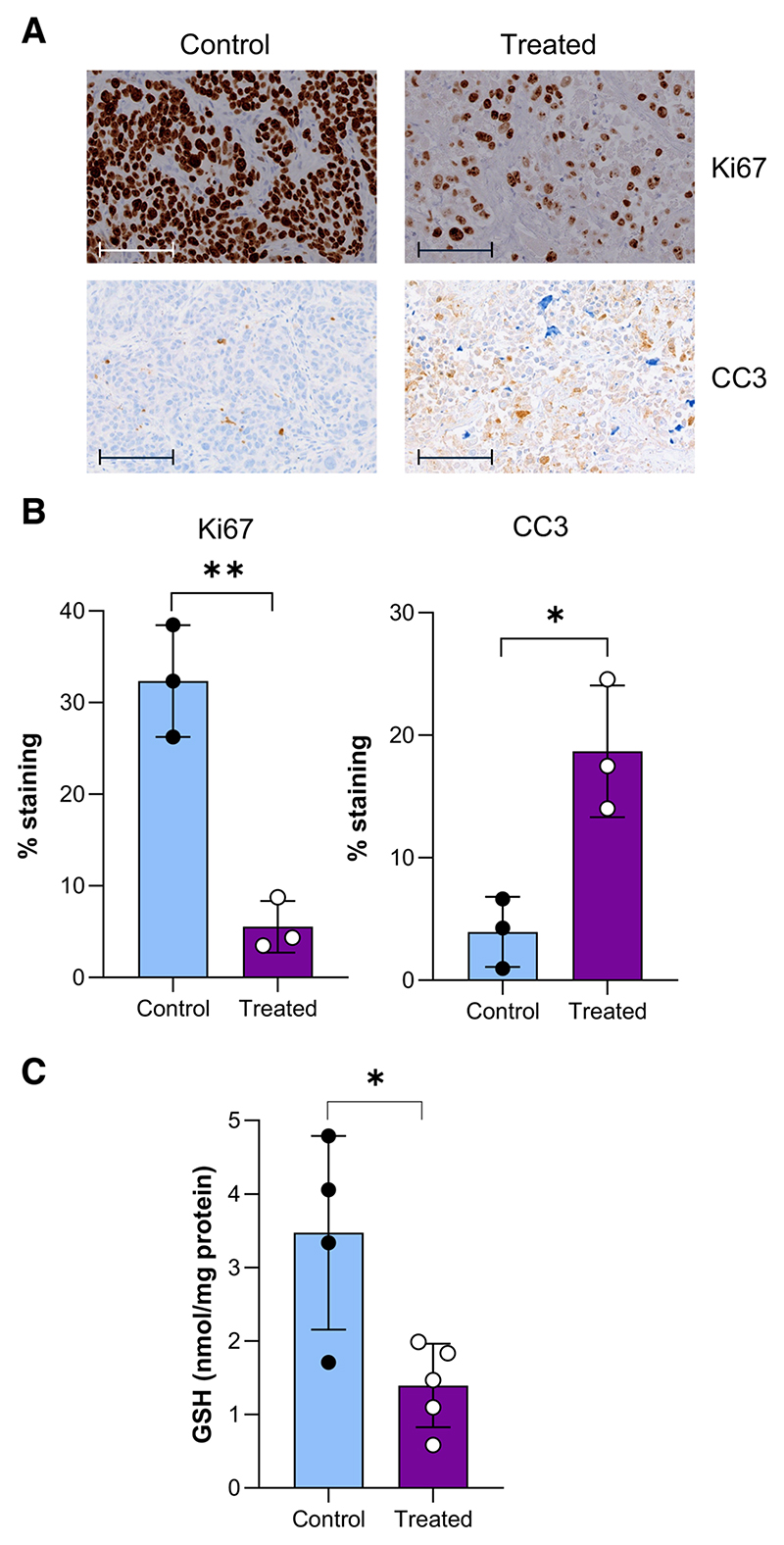
Radiotherapy treatment of FasDu tumors reduces GSH and proliferation, whilst increasing apoptotic cell death. **A** Representative IHC staining of Ki67 and cleaved caspase-3 (CC3) from ex vivo tumor samples on day 14 (scale bar = 100 μM). **B** Quantification of IHC staining. **C** Ex vivo GSH analysis of FaDU tumors.

To assess whether a decrease in [^18^F]FSPG is indicative of clinical response, tumors were excised 14 days after the start of treatment. At this time point, tumor cell proliferation was reduced by 83%, as shown by Ki67 staining (*n* = 3 individual tumors; *p* = 0.002), with the levels of apoptosis increased by 78% compared to control tumors (*n* = 3, *p* = 0.014, Fig. 3A, B). In these samples, total GSH was reduced in the radiotherapy-treated cohort compared to untreated controls, at 1.40 ± 0.57 nmol/mg protein and 3.47 ± 1.32 nmol/mg protein, respectively (*p* = 0.015, *n* = 3, Fig. 3C).

## Discussion

The identification of radioresistant tumors in HNSCC is a significant unmet clinical need, allowing chemoradiotherapy regimes to be adapted for the individual, with the potential to improve outcomes. Here, we show that [^18^F]FSPG can measure radiation response before changes in tumor size, providing an opportunity for early intervention.

In both cells and tumor-bearing mice, radiotherapy-induced oxidative stress coincided with a decrease in [^18^F]FSPG retention. This decrease in imaging signal is consistent with previously published data from our group^6,11^ following treatment with chemotherapy^7^. Whilst the mechanisms of chemotherapy-induced cell death differ from radiotherapy, both converge in the induction of DNA damage and altered redox homeostasis. In chemotherapy-treated cells, decreased cellular [^18^F]FSPG was a consequence of increased system x_c_^−^ uptake of exogenous cystine (for GSH biosynthesis), resulting in elevated [^18^F]FSPG efflux due to the transporter’s exchange mechanism^6^. We propose a similar mechanism occurs in tumors that respond to radiotherapy.

PET imaging with [^18^F]-2-fluoro-2-deoxy-D-glucose ([^18^F]FDG) is currently used in HNSCC clinical practice to determine treatment outcome. [^18^F]FDG tumor uptake, however, is affected by non-neoplastic activity, such as inflammation. Following radiotherapy treatment, there is significant peri-tumoural inflammation in surrounding nonmalignant tissues and therefore [^18^F]FDG PET imaging requires cautious interpretation. Indeed, a meta-analysis of [^18^F]FDG PET in the response assessment of HNSCC to radiotherapy treatment showed that its sensitivity for detecting residual/recurrent disease is best when conducted 10 weeks or more after completion of treatment^17^. This is too late a time point to deviate from the standard radiotherapy treatment protocols. The prognostic value of [^18^F]FDG PET during treatment remains debated^18^ and we urgently require new methods to non-invasively monitor early radiation response and resistance.

Other PET radiotracers which have been developed to monitor early radiation response in HNSCC and have been investigated in clinical studies include 3′-deoxy-3′-[^18^F]fluorothymidine ([^18^F]FLT)^19^, which provides a surrogate of cell proliferation, and [^18^F]fluoromisonidazole ([^18^F]FMISO)^20^, which is a marker of hypoxia. Of note, false-positive [^18^F]FLT uptake can occur in reactive lymph nodes^21^, creating an obstacle for radiation response assessment. [^18^F]FMISO has been extensively researched in HNSCC, and its pre-treatment uptake shows prognostic potential^22^, in addition to being able to monitor radiotherapy response in patients^23^. A major issue, however, is the lipophilicity of [^18^F]FMISO, which results in slow clearance from background tissue, producing PET images with low tissue-to-background ratios^24^ and cumbersome scanning schedules, with image acquisition frequently performed 2–4 h after radiotracer injection. As such, a significant clinical unmet need remains to develop new imaging agents, such as [^18^F]FSPG, that can sensitively measure radiation response in HNSCC.

In conclusion, we show for the first time that [^18^F]FSPG can image early response to radiation therapy. Given that [^18^F]FSPG has already been used in humans to image HNSCC, it could be rapidly rolled out to clinical studies assessing its ability to predict and monitor radiation response in human HNSCC, with the potential to change treatment planning and improve outcomes.

## Materials and methods

### Cell culture

FaDU (ATCC) was grown in Minimum Essential Medium (MEM, Thermo Fisher Scientific) supplemented with 10% fetal bovine serum (FBS, Thermo Fisher Scientific) and 100 U/mL penicillin, 100 mg/mL streptomycin (Sigma–Aldrich Ltd). Cells were maintained at 37 °C and 5% CO_2_. Mycoplasma testing was performed monthly (Eurofins).

### Radiotherapy treatment of cultured cells

1× 10^4^ and 3 × 10^5^ cells were seeded in either 96 well plates (200 μL) or 6 well plates (2 mL), respectively, 24–96 h before irradiation. Fresh media was added 1 h before radiotherapy treatments. Irradiations were performed using a SmART+ irradiator (Precision X-Ray Irradiation) using a 12 cm × 12 cm beam (225 kV, 20 mA, 0.3 mm Cu filtered) at a dose rate of 5.63 Gy/min to give a total cumulative dose of 2–10 Gy.

### Caspase-3/7 activity assay

Caspase-3/7 activity was determined using Promega’s caspase-3/7 assay according to the manufacturer’s instructions. 1 × 10^4^ cells seeded in 96 well plates were incubated for 1 h with Caspase-Glo reagent, and the enzymatic activity of caspase-3/7 was measured using a microplate luminescence reader (Promega). Data was expressed as a fold-increase in caspase-3 activity over control, unirradiated cells.

### Detection of intracellular ROS

ROS were detected in cells by CellROX Green (Thermo Fisher) according to the manufacturer’s instructions. 1 μmol/L CellROX Green reagent was added to 3 × 10^4^ cells seeded 24 h previously in 6 well plates and incubated for 30 min at 37 °C in darkness. The samples were analyzed on a BD LSR Fortessa flow cytometer (laser power 488 nm, bandpass filter 530/30 nm) with 20,000 single-cell events recorded for each experiment. Data analysis was carried out using FlowJo Software (v10.1). Post-acquisition gating was conducted using forward scattering (FS) and side scattering (SS) profiles to exclude cellular debris and cell doublets.

### In vitro analysis of total GSH

3× 10^5^ cells were seeded in 6 well plates 24 h before analysis, with total GSH determined using a luminescent assay kit (Promega) according to the manufacturer’s instructions. GSH was normalized to protein concentration (Pierce BCA protein assay kit, Thermo Fisher Scientific).

### Radiotracer production

[^18^F]FSPG radiosynthesis (GE FASTlab™) and quality control were performed according to previously published methodology^25^.

### Radiotracer uptake experiments

For cell uptake studies, 3 × 10^5^ cells were seeded in 6 well plates. 0.185 MBq [^18^F]FSPG was added in 1 mL and incubated for 60 min at 37 °C following a previously established method^26^. The radioactivity in samples was expressed as a percentage of the administered radioactivity per mg protein.

### In vivo tumor models

All animal experiments were performed under the United Kingdom Home Office Animal (Scientific Procedures) Act 1986 and received local Animal Welfare and Ethical Review Body (AWERB) approval. 1.8 × 10^6^ FaDU cancer cells in 100 μL Dulbecco’s PBS were injected subcutaneously into female Balb/c nu/nu mice aged 6–9 weeks (Charles River Laboratories). Tumor dimensions were measured using an electronic caliper and the volume was calculated using the following equation: volume = [(π/6) × *height* × *width* × *length*]. Imaging studies took place when the tumor volume reached approximately 80 mm^3^. Animals were sacrificed by cervical dislocation under anesthesia, with death confirmed by performing a secondary Schedule 1 measure under the Animals (Scientific Procedures) Act 1986.

### MicroPET imaging studies

~3.7 MBq [^18^F]FSPG was administered intravenously via the tail vein in 100 μL of PBS. The administered dose was calculated from the amount of radioactivity measured in the syringe before and after injection (both decay-corrected to the injection time). Mice were kept at 37 °C and anesthetized throughout the study (1.5–2% isoflurane). At 40 min post-injection, a 20 min PET scan was acquired (Mediso nanoScan PET/CT). Scans were attenuation-corrected from CT images (50 kVp, 480 projections) and static reconstruction was done using the Tera-Tomo 3D reconstruction algorithm (4 iterations, 6 subsets, 400–600 keV, voxel size: 0.4 mm). The resulting reconstructed images were analyzed using VivoQuant software (v. 2.5, Invicro Ltd.). Tumor volumes of interest were drawn using the CT images and expressed as a percentage of the injected dose per g of tissue (%ID/g).

### In vivo radiotherapy treatment

Animal irradiations were performed on a SmART+ irradiator (Precision X-Ray Irradiation) with SmART-ATP treatment planning software (SmART Scientific Solutions). Mice were maintained under 2% isoflurane throughout. CT-planned targeted radiotherapy treatment was delivered to the tumor using two parallel-opposed 10 mm circular beams (225 kV, 20 mA, 0.3 mm Cu filtered) at a dose rate of 5.63 Gy/min. A total of 24 Gy in eight fractions of 3 Gy was delivered over 10 days, with a 2-day break after 5 fractions. PET imaging studies were carried out on day 0 and day 7. A cohort of untreated mice, matched for tumor size at the start of the study, were also imaged on day 0 and day 7.

### Ex vivo tumor sample preparation and analysis

Immediately following sacrifice, tumor tissue was dissected, snap-frozen in liquid nitrogen, and stored at 80 °C. For GSH analysis, each tumor sample was placed into lysing matrix tubes containing 1.4-mm ceramic beads (MP Biomedicals) and 1 mL assay buffer (Promega). GSH was quantified using a luminescent assay kit (Promega) according to the manufacturer’s instructions and normalized to protein concentration. For immunohistochemical (IHC) analysis, tumors were fixed in formaldehyde and paraffin-embedded. Consecutive 5 μm tumor sections were used for the analysis of cleaved caspase-3 (CC3, 1:100, Cell Signalling) and Ki67 (1:100, abcam using a VECTOR DAB substrate kit) (Vector Laboratories) following the manufacturer’s instructions. Tissue was processed by UCL IQPath. Images were acquired using a NanoZoomer (Hamamatsu), with representative images shown. Four areas were randomly selected from the tissue sections and the percentage of staining was derived using Image J software.

### Statistical analysis

All data were expressed as the mean ± SD. Statistical significance was determined using ANOVA followed by multiple comparison correction (Tukey method; GraphPad Prism v.10.1.2).

## Supplementary Material

Supplementary data

## Data Availability

Data is available upon reasonable request to the corresponding author.
