# Author Correction: [^18^F]FSPG-PET provides an early marker of radiotherapy response in head and neck squamous cell cancer

**DOI:** 10.1038/s44303-024-00049-9

**Published:** 2024-10-01

**Authors:** Khrishanthne Sambasivan, Will E. Tyrrell, Rizwan Farooq, Jenasee Mynerich, Richard S. Edwards, Muhammet Tanc, Teresa Guerrero Urbano, Timothy H. Witney

**Affiliations:** 1 School of Biomedical Engineering and Imaging Sciences, King’s College London, London, UK; 2 Department of Clinical Oncology, Guy’s and St Thomas’ NHS Foundation Trust, London, UK; 3 Faculty of Dentistry, Oral & Craniofacial Sciences and School of Cancer & Pharmaceutical Sciences, King’s College London, London, UK

**Keywords:** Cancer imaging, Positron-emission tomography

Correction to: *npj Imaging* 10.1038/s44303-024-00038-y, published online 9 August 2024

“In this article, acknowledgement of funding from The Wellcome Trust and Cancer Research UK was omitted. The original article has now been corrected.”

