# Supplementary material for: [18F]FSPG-PET provides an early marker of radiotherapy response in head and neck squamous cell cancer
Source: Npj Imaging. Author manuscript; Available in PMC 2024 Aug 22. (PMC11315666; doi:10.1038/s44303-024-00038-y)
Supplement: Supplementary data [file EMS198052-supplement-Supplementary_data.docx]

**SUPPLEMENTAL DATA**

**[^18^F]FSPG-PET provides an early marker of radiotherapy response in head and neck squamous cell cancer**

Khrishanthne Sambasivan^1,2^ , Will E Tyrrell ^1^, Rizwan Farooq^1^, Jenasee Mynerich^1^, Richard S Edwards^1^, Muhammet Tanc^1^, Teresa Guerrero Urbano^2,3^ and Timothy H Witney^1^

**
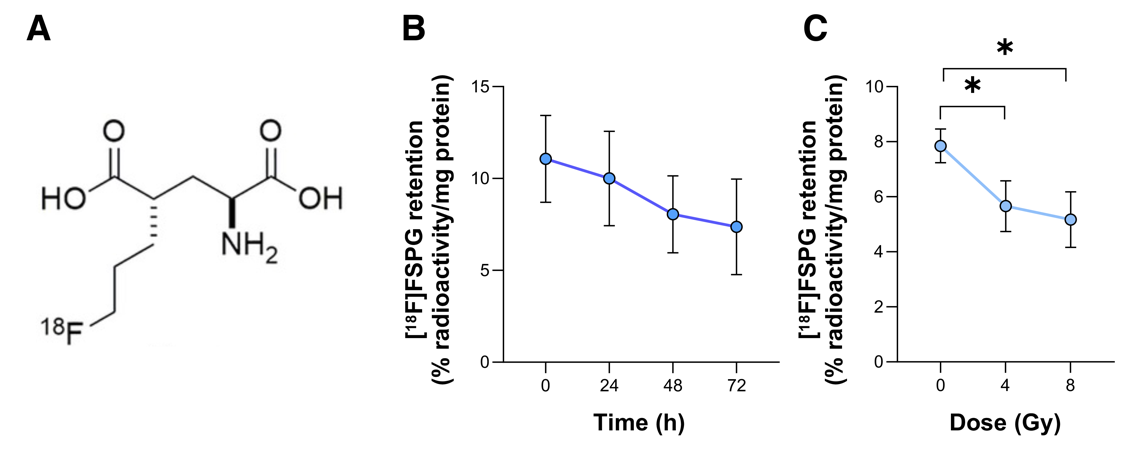
**

**Supplemental Figure 1** [^18^F]FSPG retention reduces after radiotherapy treatment. **A**, Molecular structure of [^18^F]FSPG. **B**, Time course of [^18^F]FSPG retention following 10 Gy radiotherapy. **C**, Dose-response of [^18^F]FSPG retention to radiotherapy. Data are presented as mean ± SD for *n* = 3 biological repeats.
