# Supplementary material for: [18F]FSPG-PET provides an early marker of radiotherapy response in head and neck squamous cell cancer
Source: Npj Imaging. 2024 Aug 9;2:28. doi: 10.1038/s44303-024-00038-y (PMC11315666; doi:10.1038/s44303-024-00038-y)
Supplement: Supplementary file 1 [file 44303_2024_38_MOESM1_ESM.pdf]

SUPPLEMENTAL DATA

[18F]FSPG-PET provides an early marker of radiotherapy response in head and neck squamous cell cancer

Khrishanthne Sambasivan[1,2] , Will E Tyrrell [1], Rizwan Farooq[1], Jenasee Mynerich[1], Richard S Edwards[1], Muhammet Tanc[1], Teresa Guerrero Urbano[2,3] and Timothy H Witney[1]

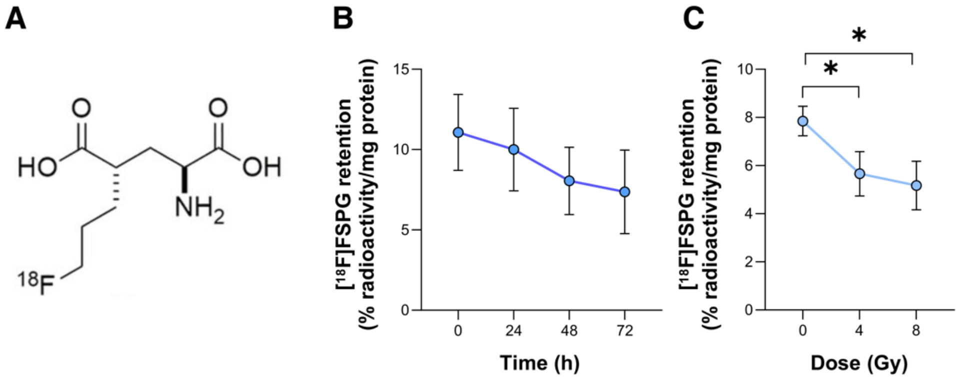

Supplemental Figure 1 [^{18}F]FSPG retention reduces after radiotherapy treatment. **A**, Molecular structure of [^{18}F]FSPG. **B**, Time course of [^{18}F]FSPG retention following 10 Gy radiotherapy. **C**, Dose-response of [^{18}F]FSPG retention to radiotherapy. Data are presented as mean ± SD for *n* = 3 biological repeats.
